# Evaluating the impact of metabolic indicators and scores on cardiovascular events using machine learning

**DOI:** 10.1186/s13098-025-01753-1

**Published:** 2025-05-30

**Authors:** Guanmou Li, Cheng Luo, Teng Ge, Kunyang He, Miao Zhang, Jinlin Hu, Baoshi Zheng, Rongjun Zou, Xiaoping Fan

**Affiliations:** 1 State Key Laboratory of Traditional Chinese Medicine Syndrome, State Key Laboratory of Dampness Syndrome of Chinese Medicine, Department of Cardiovascular Surgery, Guangdong Provincial Hospital of Chinese Medicine, The Second Affiliated Hospital of Guangzhou University of Chinese Medicine, The Second Clinical College of Guangzhou University of Chinese Medicine, Guangzhou, 510120 Guangdong China; 2 Guangdong Provincial Key Laboratory of TCM Emergency Research, Guangzhou, 510120 Guangdong China; 3 Zhu Jiang Hospital, Southern Medical University, Guangzhou, 510260 China; 4 Department of Cardiovascular Surgery, The First Affiliated Hospital of Guangxi Medical University, Nanning, 530021 China; 5 Department of Pharmacology, School of Pharmaceutical, Guangzhou University of Chinese Medicine, No. 232 Waihuan Dong Rd., Guangzhou University Town, Panyu District, Guangzhou, 510000 China

**Keywords:** Heart failure, Myocardial infarction, Angina pectoris, Coronary disease, Metabolism

## Abstract

Cardiovascular diseases such as coronary artery disease, myocardial infarction, and heart failure impact millions of people annually globally and are a major cause of disease and death. This study explores the predictive capabilities of novel metabolic indices (TyG, HOMA-IR, TG/HDL-C, and VAI) for major adverse cardiovascular events (MACE) and analyzes their relationships with diabetes and cardiovascular risks. Using data from the National Health and Nutrition Examination Survey (NHANES) spanning from 2003 to 2018, we applied multiple machine learning algorithms to evaluate nine metabolic indicators including cholesterol levels, triglycerides, insulin, and waist circumference. Through cross-validation to validate model performance, the XGBoost algorithm demonstrated the most accurate performance in predicting cardiovascular outcomes, particularly for diseases like angina and heart failure. Additionally, SHAP value analysis confirmed the critical roles of waist circumference and METS-IR in predicting adverse cardiovascular events. Furthermore, we employed 100 machine learning algorithms to calculate the AUC values of metabolic indicators in predicting AP, CHD, HF, and MI, showing that METS-IR had the greatest contribution in these aspects. This research highlights the significance of metabolic indices in stratifying cardiovascular risks and presents potential avenues for targeted preventive strategies.

**Supplementary Information:**

The online version contains supplementary material available at 10.1186/s13098-025-01753-1.

## Introduction

Major Adverse Cardiovascular Events (MACE) refers to cardiovascular complications occurring in patients with myocardial injury after non-cardiac surgery (MINS). These complications include coronary disease, myocardial infarction, myocardial ischemia, and heart failure [1]. Angina pectoris is chest pain caused by myocardial ischemia, typically aggravated by physical activity or emotional stress, lasting for more than 30 to 60 s, and relieved by rest and nitroglycerin use. It is estimated that approximately 10 million people in the United States suffer from angina pectoris, with over 500,000 new cases each year [2]. Coronary disease (CHD) is a cardiovascular disease caused by insufficient blood supply to the myocardium due to coronary artery disease and is one of the leading causes of death worldwide. As of 2020, over 10 million people die from coronary disease each year globally [3]. Myocardial infarction (MI) is caused by interrupted blood flow to the myocardial tissue, resulting in tissue death. According to statistics from the American Heart Association, approximately 805,000 Americans experience myocardial infarction each year, with around 605,000 as first-time cases and 200,000 as recurrent myocardial infarctions. Additionally, approximately 122,000 people die from myocardial infarction annually, accounting for 14% of total deaths [4, 5]. Heart failure (HF) is a disease where the heart is unable to pump blood effectively enough to meet the body's needs, leading to symptoms such as fatigue, difficulty breathing, and edema. In the United States, heart failure is a common and increasingly severe disease. It is estimated that approximately 6 million Americans suffer from heart failure, with approximately 1 million people hospitalized for heart failure each year [6].

The development of diabetes is closely related to the occurrence of adverse cardiovascular events. Currently, the main metabolic score for evaluating diabetes is HOMA-IR (Homeostasis Model Assessment-Insulin Resistance Index) [7]. However, four new indicators—VAI (Visceral Adiposity Index), TyG (Triglyceride and Glucose), TG/HDL-C (Ratio of triglyceride to high-density lipoprotein cholesterol), and METS-IR (metabolic score for insulin resistance)—play key roles in predicting the onset of diabetes and are expected to replace HOMA-IR as the key scoring system for diagnosing diabetes [8]. At the same time, in adverse cardiovascular events [9], patients with high levels of VAI have a significantly increased risk of angina [10–12]; when HOMA-IR exceeds 3.59, an increase in HOMA-IR can significantly raise the mortality rate of patients with coronary heart disease combined with hypertension, and the elevation of HOMA-IR also significantly increases the risk of atherosclerosis [13, 14]; studies have also shown that the TyG index is positively correlated with the incidence of myocardial infarction in American adults, serving as a key metabolic score for predicting myocardial infarction occurrence [15]. Recent research has also indicated that a high TyG index may induce exacerbated inflammatory responses and oxidative stress, which play important roles in the progression of heart failure [16]. Meanwhile, TG/HDL-C is significantly associated with the five-year mortality risk of heart failure patients [17]. A high TG/HDL-C ratio reflects lipid metabolism disorders and abnormal blood lipid levels, which may increase the risk of endothelial dysfunction and the occurrence of atherosclerosis [18]. However, there has been no literature reported on which of the five metabolic scores—HOMA-IR, VAI, TyG, TG/HDL-C, and METS-IR—has an impact on adverse cardiovascular events.

In this study, we used data from the National Health and Nutrition Examination Survey (NHANES, 2003–2018) to investigate the relationship between metabolic indicators and scores and angina pectoris, coronary disease, myocardial infarction, and heart failure in major adverse cardiovascular events. We employed six binary classification machine learning algorithms to analyze angina pectoris, coronary disease, myocardial infarction, and heart failure, and selected the most suitable binary classification machine learning algorithm for evaluating various metabolic indicators and score models. Additionally, based on Shapley Additive explanations, we provided explanations for the model that best evaluated major adverse cardiovascular events and sought out the most crucial metabolic indicators and scores in these events.The purpose of this study is to explore the most critical metabolic scores affecting adverse cardiovascular events through a variety of machine learning algorithms.

## Method and materials

### Study participants

The NHANES study conducted interviews and physical examinations on the US population using a variety of survey strategies. Our study included eight consecutive cycles of NHANES from 2003 to 2018. The exclusion criteria were as follows: (1) participants with uncertain status of angina pectoris, coronary disease, myocardial infarction, and heart failure based on the NHANES questionnaire (indicated by a response of 7 or 9); (2) missing values for the included metabolic indicators (see Supplementary Fig. 1–4 for details); (3) participants under the age of 20 were excluded.

### Data collection

Demographic and social characteristics of the participants were collected from the US population data. These characteristics included gender, age (in years), race/ethnicity, education level (college or above, high school or equivalent, below high school), and poverty income ratio (PIR), etc. In this study, to minimize the impact of factors other than metabolic indicators on major adverse cardiovascular events, we only included four essential demographic and social characteristics: gender, age, race, and PIR.

Our study also included nine metabolic indicators in human blood, including TC (total cholesterol), AIC (glycated hemoglobin), TG (triglycerides), LDL-C (low-density lipoprotein cholesterol), INSULIN (insulin), HDL-C (high-density lipoprotein cholesterol), PFG (fasting plasma glucose), BMI (body mass index), and WAIST (waist circumference). Blood samples were processed, stored, and transported to the Fairview Medical Center laboratory at the University of Minnesota in Minneapolis, Minnesota, for analysis. In addition, we included five metabolic scores closely related to the cardiovascular system in human blood to optimize the predictive and explanatory power of the model: VAI, TyG, TG/HDL-C, METS-IR, and HOMA-IR. We also selected participants who reported being diagnosed or informed by a doctor of angina pectoris, coronary disease, myocardial infarction, and heart failure as the binary outcome variables for machine learning.

### Machine learning

In this study, multiple machine learning algorithms were applied to evaluate the predictive models of angina pectoris, coronary disease, myocardial infarction, and heart failure composed of various metabolic indicators and scores. The research data were divided into an 80% training set and a 20% testing set. In the binary logistic regression algorithm, we used maximum likelihood estimation to fit the model, determine the relationship between independent and dependent variables, and predict the classification probability of samples by mapping the output of the linear function into a probability range [19]. We also employed the AdaBoost algorithm as an ensemble learning method, enhancing the accuracy of classification by combining multiple weak learners, such as decision trees. This algorithm focuses more on misclassified samples, thus optimizing the overall model performance [20]. The decision tree algorithm is used to describe the relationship between features and the target variable, obtaining clear classification rules by continuously dividing the feature space. This model is intuitive and suitable for decision-making under specific conditions [21]. XGBoost, as an optimized gradient boosting algorithm, possesses unique advantages in improving model prediction accuracy and controlling overfitting. We used this algorithm for model construction and enhanced the generalization ability of the model through regularization techniques [22]. We also employed the random forest algorithm, constructing multiple decision trees by randomly selecting and sampling features and the sample set, and then combining them into a powerful ensemble model to predict the binary models of angina pectoris, coronary disease, myocardial infarction, and heart failure separately [23]. Additionally, the Gaussian Naive Bayes classification (GNB) algorithm was used to estimate the prior probability of each category, as well as the mean and variance of each feature. By calculating the mean and variance of each feature under different categories, the Gaussian distribution parameters of the features in the binary models of angina pectoris, coronary disease, myocardial infarction, and heart failure can be obtained. These parameters can be used to calculate the posterior probability of a sample belonging to a certain category given a specific feature condition [24].

The training process of all models was conducted using cross-validation methods to ensure the reliability and robustness of the results. Furthermore, we evaluated the classification performance of different models, focusing on metrics such as accuracy, recall, and F1 score, to select the best classification model for further analysis. After combining the discriminative features of each model, we selected the models that were most suitable for identifying angina pectoris, coronary disease, myocardial infarction, and heart failure. We used SHAP values to explain the selected models, incorporating the risk variables related to angina pectoris, coronary disease, myocardial infarction, and heart failure from the participants spanning from 2003 to 2018 in the NHANES questionnaire data.

We also utilized 100 combinations of eight machine learning algorithms: Enet, SVM, glm, RF, GBM, plsRglmmodel, Ridge, and Lasso, to evaluate the predictive effectiveness of the metabolic indicators and scores we established for AP, CHD, HF, and MI. In this study, we employed a clinical sample dataset with AP, CHD, HF, and MI as the target variables. Based on previous research and expert recommendations, we constructed a series of metabolic indicators and scores to predict the occurrence of different cardiovascular events. Subsequently, we built a hundred different predictive models by utilizing various combinations of these machine learning algorithms and trained and optimized the models using the training dataset. Finally, we evaluated the performance of these models using a testing dataset, measuring the AUC (Area Under the Curve). These evaluation results will help determine which metabolic indicators and scores have the best predictive effectiveness for AP, CHD, HF, and MI.

## Results

### Evaluation of the influence of metabolic indicators and scores on angina pectoris using binary classification machine learning algorithms and machine learning algorithm explanations

**Fig. 1:**
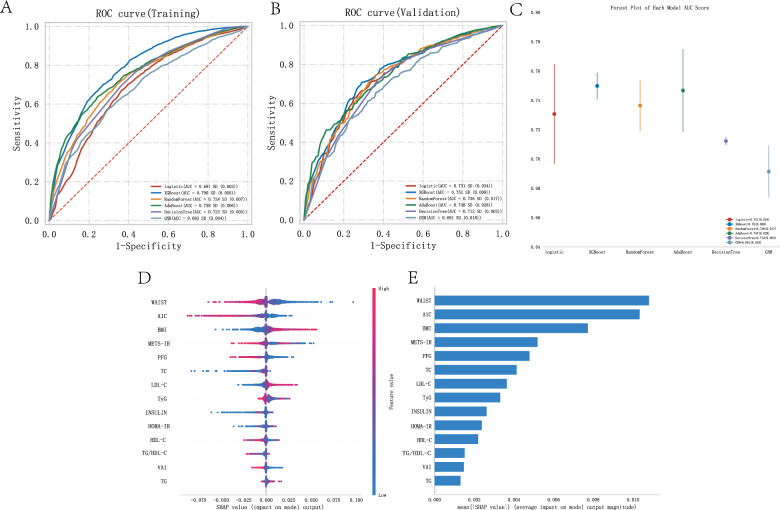
Machine learning model evaluation of various metabolic indicators and scores in the angina pectoris population. **A** AUC results graph of the training set. **B** AUC results graph of the testing set. **C** Forest plot of AUC scores. **D** Summary of the shap model of the XGBoost algorithm. **E** Importance ranking results of the shap model of the XGBoost algorithm

In a representative sample of 16,299 US adults, 48.37% were male (= 7,883) and 51.63% were female (= 8,416). Angina pectoris was identified in 435 patients, accounting for 2.67% of the study population. The age of the angina pectoris group ranged from 60 to 77 years, with an average PIR of 1.79. Among the patients, 11% were Mexican American, 7% were Other Hispanic, 62% were Non-Hispanic White, 14% were Non-Hispanic Black, and 6% were Other Race Including Multi Racial. In the blood of the angina pectoris group, the average concentrations were as follows: TC (173 mg/dL), A1 C (5.9 mg/dL), TG (119 mg/dL), LDL-C (97 mg/dL), INSULIN (11.28 uU/mL), HDL-C (1.22 mg/dL), PFG (110 mg/dL), BMI (29.54 kg/m2), WAIST (105.10 cm), VAI (160.47), TyG (8.83), TG/HDL-C (95.88), METS-IR (1.79), and HOMA-IR (3.14) (Table 1). Figure 1A shows the ROC curves of each model based on the training data, providing a comparative analysis of model performance and their AUC values. The AUC for the logistic algorithm was 0.691, XGBoost algorithm was 0.790, RandomForest algorithm was 0.734, AdaBoost algorithm was 0.758, DecisionTree algorithm was 0.723, and GNB algorithm was 0.683. These ROC curves depict the sensitivity and specificity of various metabolic indicators and score models in influencing angina pectoris, serving as key benchmarks for evaluating the effects of different algorithms based on these indicators and scores. Figure 1B extends this evaluation to the validation data, showing the ROC curves that reflect the generalization ability of the models in unseen data. The AUC for the logistic algorithm was 0.731, XGBoost algorithm was 0.751, RandomForest algorithm was 0.736, AdaBoost algorithm was 0.748, DecisionTree algorithm was 0.712, and GNB algorithm was 0.691. The AUC values provide a quantitative assessment of the accuracy of each model, which is crucial for validating their predictive ability in clinical and research settings. Figure 1C compares the AUC scores of different ML models in the validation data using a forest plot. Each point in the plot represents the median AUC value of a model, and the error bars represent the confidence intervals, emphasizing the performance differences between the models. In both the training and validation sets, the XGBoost algorithm achieved the highest AUC value, demonstrating the critical role played by various metabolic indicators and scores in influencing angina pectoris. In Fig. 1D and E, we subsequently built the SHAP models using the XGBoost algorithm, showing that waist circumference had the greatest impact on angina pectoris in the metabolic models. METS-IR had the largest impact among the five metabolic scores (METS-IR, TyG, HOMA-IR, TG/HDL-C, and VAI) on angina pectoris.

**Table 1:** Baseline characteristics of participants associated with angina (AP)

Variable	AP, N = 435^1^	CON, N = 15,864^1^	p-value^2^
AGE, Median (IQR)	68.00 (60.00–77.00)	48.00 (34.00–63.00)	< 0.001
PIR, Median (IQR)	1.79 (1.09–3.14)	2.16 (1.14–4.10)	< 0.001
TC, Median (IQR)	173.00 (145.00–210.00)	190.00 (164.00–218.00)	< 0.001
A1 C, Median (IQR)	5.90 (5.50–6.60)	5.50 (5.20–5.80)	< 0.001
TG, Median (IQR)	119.00 (86.00–165.00)	103.00 (72.00–151.00)	< 0.001
LDL-C, Median (IQR)	97.00 (72.50–123.00)	112.00 (89.00–136.00)	< 0.001
INSULIN, Median (IQR)	11.28 (6.67–19.95)	9.38 (5.89–15.44)	< 0.001
HDL-C, Median (IQR)	1.22 (1.03–1.50)	1.34 (1.11–1.63)	< 0.001
PFG, Median (IQR)	110.00 (99.00–130.00)	100.00 (92.20–110.00)	< 0.001
BMI, Median (IQR)	29.54 (26.13–34.09)	27.82 (24.20–32.30)	< 0.001
WAIST, Median (IQR)	105.10 (96.30–115.85)	97.50 (87.50–108.40)	< 0.001
VAI, Median (IQR)	160.47 (101.56–241.89)	125.80 (76.91–209.69)	< 0.001
TyG, Median (IQR)	8.83 (8.49–9.27)	8.57 (8.17–9.01)	< 0.001
TG/HDL-C, Median (IQR)	95.88 (61.31–148.97)	75.91 (46.23–125.00)	< 0.001
METS-IR, Median (IQR)	1.79 (1.69–1.91)	1.71 (1.61–1.81)	< 0.001
HOMA-IR, Median (IQR)	3.14 (1.79–6.06)	2.38 (1.42–4.16)	< 0.001
Gender, n (%)			< 0.001
Male	253 (58)	7,630 (48)	
Female	182 (42)	8,234 (52)	
Race, n (%)			< 0.001
Mexican American	50 (11)	2,550 (16)	
Other Hispanic	29 (7)	1,374 (9)	
Non-Hispanic White	268 (62)	7,091 (45)	
Non-Hispanic Black	62 (14)	3,186 (20)	
Other Race (Multi-Racial)	26 (6)	1,663 (10)	

^1^Median (IQR) or Frequency (%)

^2^Wilcoxon rank sum test; Pearson's Chi-squared test

### Evaluation of the influence of metabolic indicators and scores on coronary disease using binary classification machine learning algorithms and machine learning algorithm explanations

**Fig. 2:**
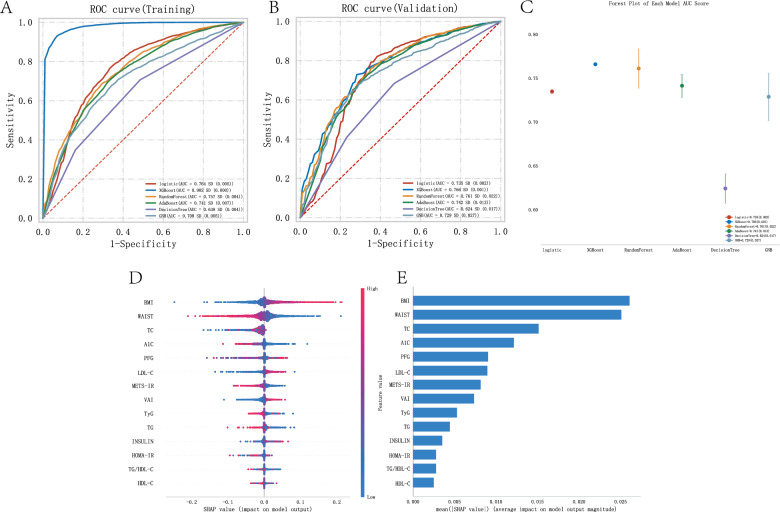
Machine learning model evaluation of various metabolic indicators and scores in the coronary disease population. **A** AUC results graph of the training set. **B** AUC results graph of the testing set. **C** Forest plot of AUC scores. **D** Summary of the shap model of the XGBoost algorithm. **E** Importance ranking results of the shap model of the XGBoost algorithm

In a representative sample of 16,287 US adults, 48.33% were male (= 7,871) and 51.67% were female (= 8,416). coronary disease was identified in 679 patients, accounting for 3.73% of the study population. The age of the coronary disease group ranged from 63 to 79 years, with an average PIR of 2.15. Among the patients, 9.1% were Mexican American, 6.7% were Other Hispanic, 67% were Non-Hispanic White, 11% were Non-Hispanic Black, and 6.6% were Other Race Including Multi Racial. In the blood of the coronary disease group, the average concentrations were as follows: TC (166 mg/dL), A1 C (5.9 mg/dL), TG (115 mg/dL), LDL-C (90 mg/dL), INSULIN (11.19 uU/mL), HDL-C (1.22 mg/dL), PFG (111 mg/dL), BMI (28.59 kg/m2), WAIST (104.10 cm), VAI (157.53), TyG (8.81), TG/HDL-C (96.49), METS-IR (1.82), and HOMA-IR (3.09) (Table 2). The ROC curves of each model based on the training data, providing a comparative analysis of model performance and their AUC values (Fig. 2A). The AUC for the logistic algorithm was 0.764, XGBoost algorithm was 0.982, RandomForest algorithm was 0.757, AdaBoost algorithm was 0.741, DecisionTree algorithm was 0.638, and GNB algorithm was 0.708. These ROC curves depict the sensitivity and specificity of various metabolic indicators and score models in influencing coronary disease, serving as key benchmarks for evaluating the effects of different algorithms based on these indicators and scores. Figure 2B extends this evaluation to the validation data, showing the ROC curves that reflect the generalization ability of the models in unseen data. The AUC for the logistic algorithm was 0.735, XGBoost algorithm was 0.766, RandomForest algorithm was 0.761, AdaBoost algorithm was 0.742, DecisionTree algorithm was 0.624, and GNB algorithm was 0.729. The AUC values provide a quantitative assessment of the accuracy of each model, which is crucial for validating their predictive ability in clinical and research settings. Figure 2C compares the AUC scores of different ML models in the validation data using a forest plot. Each point in the plot represents the median AUC value of a model, and the error bars represent the confidence intervals, emphasizing the performance differences between the models. In both the training and validation sets, the XGBoost algorithm achieved the highest AUC value, demonstrating the critical role played by various metabolic indicators and scores in influencing coronary disease. In Fig. 2D and E, we subsequently built the SHAP models using the XGBoost algorithm, showing that BMI had the greatest impact on coronary disease in the metabolic models. METS-IR had the largest impact among the five metabolic scores (METS-IR, TyG, HOMA-IR, TG/HDL-C, and VAI) on coronary disease.

**Table 2:** Baseline characteristics of participants associated with coronary disease (CHD)

Variable	CHD, N = 679^1^	CON, N = 15,608^1^	p-value^2^
AGE, Median (IQR)	70.00 (63.00–79.00)	48.00 (33.00–63.00)	< 0.001
PIR, Median (IQR)	2.15 (1.20–3.92)	2.15 (1.13–4.09)	0.7
TC, Median (IQR)	166.00 (141.00–198.00)	191.00 (165.00–218.00)	< 0.001
A1 C, Median (IQR)	5.90 (5.50–6.50)	5.50 (5.20–5.80)	< 0.001
TG, Median (IQR)	115.00 (84.50–170.00)	103.00 (72.00–150.00)	< 0.001
LDL-C, Median (IQR)	90.00 (69.50–115.00)	112.00 (90.00–137.00)	< 0.001
INSULIN, Median (IQR)	11.19 (6.68–18.90)	9.35 (5.89–15.41)	< 0.001
HDL-C, Median (IQR)	1.22 (1.03–1.50)	1.34 (1.11–1.66)	< 0.001
PFG, Median (IQR)	111.00 (99.00–129.00)	99.60 (92.00–109.00)	< 0.001
BMI, Median (IQR)	28.59 (25.22–32.61)	27.82 (24.20–32.30)	0.001
WAIST, Median (IQR)	104.10 (94.85–115.00)	97.30 (87.30–108.20)	< 0.001
VAI, Median (IQR)	157.53 (93.82–240.34)	125.38 (76.79–209.08)	< 0.001
TyG, Median (IQR)	8.81 (8.42–9.27)	8.57 (8.16–9.00)	< 0.001
TG/HDL-C, Median (IQR)	96.49 (59.75–149.53)	75.55 (46.20–124.43)	< 0.001
METS-IR, Median (IQR)	1.82 (1.71–1.93)	1.70 (1.61–1.81)	< 0.001
HOMA-IR, Median (IQR)	3.09 (1.74–5.80)	2.37 (1.42–4.14)	< 0.001
*Gender, n (%)*			< 0.001
Male	463 (68)	7,408 (47)	
Female	216 (32)	8,200 (53)	
*Race, n (%)*			< 0.001
Mexican American	62 (9.1)	2,531 (16)	
Other Hispanic	44 (6.7)	1,360 (8.7)	
Non-Hispanic White	454 (67)	6,896 (44)	
Non-Hispanic Black	74 (11)	3,177 (20)	
Other Race—Including Multi-Racial	45 (6.6)	1,644 (11)	

^1^Median (IQR) or Frequency (%)

^2^Wilcoxon rank sum test; Pearson's Chi-squared test

### Evaluation of the influence of metabolic indicators and scores on myocardial infarction using binary classification machine learning algorithms and machine learning algorithm explanations

**Fig. 3:**
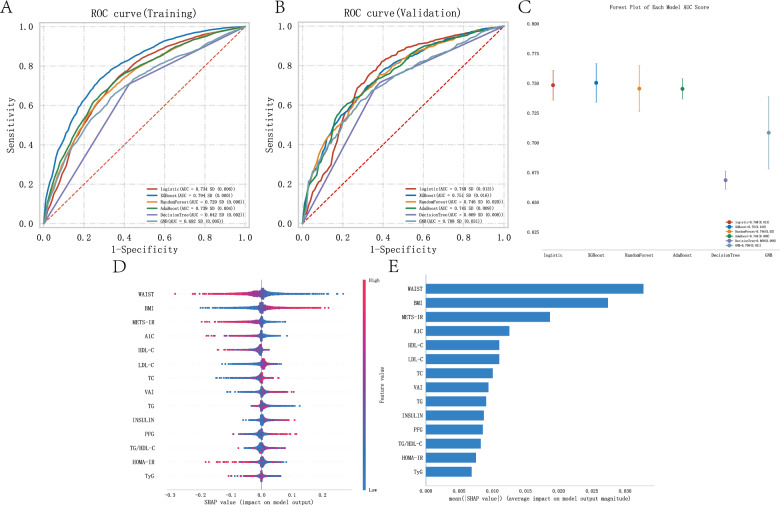
Machine learning model evaluation of various metabolic indicators and scores in the myocardial infarction population. **A** AUC results graph of the training set. **B** AUC results graph of the testing set. **C** Forest plot of AUC scores. **D** Summary of the shap model of the XGBoost algorithm. **E** Importance ranking results of the shap model of the XGBoost algorithm

In a representative sample of 16,324 US adults, 48.37% were male (= 7,896) and 51.63% were female (= 8,428). Myocardial infarction was identified in 712 patients, accounting for 4.23% of the study population. The age of the myocardial infarction group ranged from 60 to 77 years, with an average PIR of 1.65. Among the patients, 8.8% were Mexican American, 7.2% were Other Hispanic, 60% were Non-Hispanic White, 17% were Non-Hispanic Black, and 7% were Other Race Including Multi Racial. In the blood of the myocardial infarction group, the average concentrations were as follows: TC (171 mg/dL), A1 C (5.95 mg/dL), TG (114 mg/dL), LDL-C (92 mg/dL), INSULIN (10.82 uU/mL), HDL-C (1.22 mg/dL), PFG (109 mg/dL), BMI (29.05 kg/m2), WAIST (104.45 cm), VAI (149.56), TyG (8.78), TG/HDL-C (92.33), METS-IR (1.80), and HOMA-IR (3.08) (Table 3).Here, the ROC curves of each model based on the training data, providing a comparative analysis of model performance and their AUC values (Fig. 3A). The AUC for the logistic algorithm was 0.734, XGBoost algorithm was 0.794, RandomForest algorithm was 0.729, AdaBoost algorithm was 0.739, DecisionTree algorithm was 0.642, and GNB algorithm was 0.692. These ROC curves depict the sensitivity and specificity of various metabolic indicators and score models in influencing myocardial infarction, serving as key benchmarks for evaluating the effects of different algorithms based on these indicators and scores. Figure 3B extends this evaluation to the validation data, showing the ROC curves that reflect the generalization ability of the models in unseen data. The AUC for the logistic algorithm was 0.748, XGBoost algorithm was 0.751, RandomForest algorithm was 0.746, AdaBoost algorithm was 0.745, DecisionTree algorithm was 0.669, and GNB algorithm was 0.708. The AUC values provide a quantitative assessment of the accuracy of each model, which is crucial for validating their predictive ability in clinical and research settings. Figure 3C compares the AUC scores of different ML models in the validation data using a forest plot. Each point in the plot represents the median AUC value of a model, and the error bars represent the confidence intervals, emphasizing the performance differences between the models. In both the training and validation sets, the XGBoost algorithm achieved the highest AUC value, demonstrating the critical role played by various metabolic indicators and scores in influencing myocardial infarction. In Fig. 3D and E, we subsequently built the SHAP models using the XGBoost algorithm, showing that waist circumference had the greatest impact on myocardial infarction in the metabolic models. METS-IR had the largest impact among the five metabolic scores (METS-IR, TyG, HOMA-IR, TG/HDL-C, and VAI) on myocardial infarction.

**Table 3:** Baseline characteristics of participants associated with myocardial infarction (MI)

Variable	MI, N = 712^1^	CON, N = 15,612^1^	p-value^2^
AGE, Median (IQR)	69.00 (60.00–77.00)	48.00 (33.00–63.00)	< 0.001
PIR, Median (IQR)	1.65 (1.02–3.12)	2.18 (1.14–4.11)	< 0.001
TC, Median (IQR)	171.00 (143.75–200.00)	190.00 (165.00–218.00)	< 0.001
A1 C, Median (IQR)	5.90 (5.50–6.50)	5.50 (5.20–5.80)	< 0.001
TG, Median (IQR)	114.00 (82.00–166.00)	103.00 (72.00–150.25)	< 0.001
LDL-C, Median (IQR)	92.00 (72.00–118.00)	112.00 (90.00–137.00)	< 0.001
INSULIN, Median (IQR)	10.82 (6.84–19.29)	9.34 (5.88–15.40)	< 0.001
HDL-C, Median (IQR)	1.22 (1.03–1.53)	1.34 (1.11–1.66)	< 0.001
PFG, Median (IQR)	109.00 (99.00–127.00)	99.75 (92.00–109.00)	< 0.001
BMI, Median (IQR)	29.05 (25.19–32.97)	27.81 (24.20–32.30)	< 0.001
WAIST, Median (IQR)	104.45 (94.58–115.33)	97.40 (87.30–108.20)	< 0.001
VAI, Median (IQR)	149.56 (92.32–241.98)	125.57 (76.87–209.43)	< 0.001
TyG, Median (IQR)	8.78 (8.40–9.26)	8.57 (8.16–9.00)	< 0.001
TG/HDL-C, Median (IQR)	92.33 (58.79–150.12)	75.73 (46.21–124.65)	< 0.001
METS-IR, Median (IQR)	1.80 (1.70–1.92)	1.70 (1.61–1.81)	< 0.001
HOMA-IR, Median (IQR)	3.08 (1.81–5.70)	2.37 (1.42–4.14)	< 0.001
*Gender, n (%)*			< 0.001
Male	468 (66)	7,428 (48)	
Female	244 (34)	8,184 (52)	
*Race, n (%)*			< 0.001
Mexican American	63 (8.8)	2,540 (16)	
Other Hispanic	51 (7.2)	1,357 (8.7)	
Non-Hispanic White	430 (60)	6,937 (44)	
Non-Hispanic Black	123 (17)	3,130 (20)	
Other Race—Including Multi-Racial	45 (7)	1,648 (11)	

^1^Median (IQR) or Frequency (%)

^2^Wilcoxon rank sum test; Pearson's Chi-squared test

### Evaluation of the influence of metabolic indicators and scores on heart failure using binary classification machine learning algorithms and machine learning algorithm explanations

**Fig. 4:**
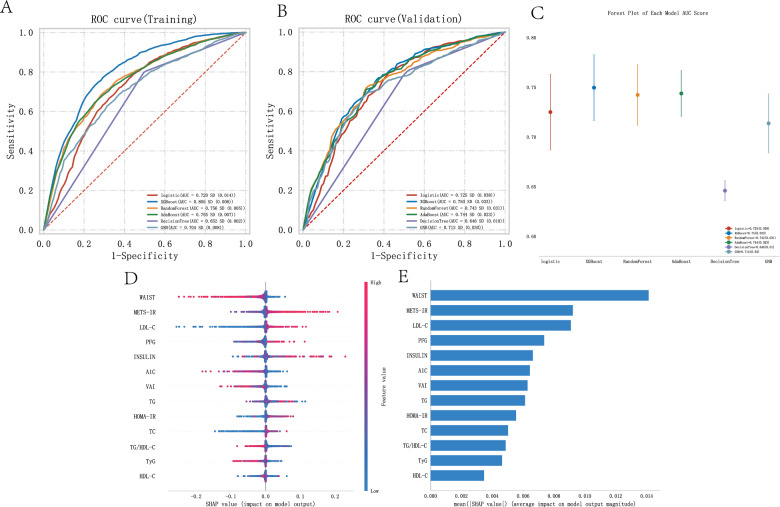
Machine learning model evaluation of various metabolic indicators and scores in the heart failure population. **A** AUC results graph of the training set. **B** AUC results graph of the testing set. **C** Forest plot of AUC scores. **D** Summary of the shap model of the XGBoost algorithm. **E** Importance ranking results of the shap model of the XGBoost algorithm

In a representative sample of 16,305 US adults, 48.33% were male (= 7,881) and 51.63% were female (= 8,424). Heart failure was identified in 500 patients, accounting for 3.07% of the study population. The age of the heart failure group ranged from 60 to 78 years, with an average PIR of 1.57. Among the patients, 8.4% were Mexican American, 7.2% were Other Hispanic, 58% were Non-Hispanic White, 22% were Non-Hispanic Black, and 4.4% were Other Race Including Multi Racial. In the blood of the heart failure group, the average concentrations were as follows: TC (170 mg/dL), A1 C (5.95 mg/dL), TG (115 mg/dL), LDL-C (93.5 mg/dL), INSULIN (11.4 uU/mL), HDL-C (1.22 mg/dL), PFG (109 mg/dL), BMI (30.1 kg/m2), WAIST (107.4 cm), VAI (163.10), TyG (8.79), TG/HDL-C (94.81), METS-IR (1.82), and HOMA-IR (3.11) (Table 4). Similarly, the ROC curves of each model based on the training data, providing a comparative analysis of model performance and their AUC values (Fig. 4A). The AUC for the logistic algorithm was 0.720, XGBoost algorithm was 0.805, RandomForest algorithm was 0.756, AdaBoost algorithm was 0.755, DecisionTree algorithm was 0.652, and GNB algorithm was 0.704. These ROC curves depict the sensitivity and specificity of various metabolic indicators and score models in influencing heart failure, serving as key benchmarks for evaluating the effects of different algorithms based on these indicators and scores. Figure 4B extends this evaluation to the validation data, showing the ROC curves that reflect the generalization ability of the models in unseen data. The AUC for the logistic algorithm was 0.725, XGBoost algorithm was 0.750, RandomForest algorithm was 0.743, AdaBoost algorithm was 0.744, DecisionTree algorithm was 0.646, and GNB algorithm was 0.713. The AUC values provide a quantitative assessment of the accuracy of each model, which is crucial for validating their predictive ability in clinical and research settings. Figure 4C compares the AUC scores of different ML models in the validation data using a forest plot. Each point in the plot represents the median AUC value of a model, and the error bars represent the confidence intervals, emphasizing the performance differences between the models. In both the training and validation sets, the XGBoost algorithm achieved the highest AUC value, demonstrating the critical role played by various metabolic indicators and scores in influencing heart failure. In Fig. 4D and E, we subsequently built the SHAP models using the XGBoost algorithm, showing that waist circumference had the greatest impact on heart failure in the metabolic models. METS-IR had the largest impact among the five metabolic scores (METS-IR, TyG, HOMA-IR, TG/HDL-C, and VAI) on heart failure.

**Table 4:** Baseline characteristics of participants associated with heart failure (HF)

Variable	HF, N = 500^1^	CON, N = 15,805^1^	p-value^2^
AGE, Median (IQR)	70.00 (60.00–78.00)	48.00 (34.00–63.00)	< 0.001
PIR, Median (IQR)	1.57 (1.02–2.89)	2.17 (1.14–4.12)	< 0.001
TC, Median (IQR)	170.00 (142.00–202.00)	190.00 (164.00–218.00)	< 0.001
A1 C, Median (IQR)	5.95 (5.50–6.63)	5.50 (5.20–5.80)	< 0.001
TG, Median (IQR)	115.00 (81.75–166.25)	103.00 (72.00–150.00)	< 0.001
LDL-C, Median (IQR)	93.50 (70.75–121.00)	112.00 (90.00–136.00)	< 0.001
INSULIN, Median (IQR)	11.40 (6.40–22.71)	9.36 (5.89–15.41)	< 0.001
HDL-C, Median (IQR)	1.22 (1.01–1.50)	1.34 (1.11–1.63)	< 0.001
PFG, Median (IQR)	109.00 (98.00–130.00)	100.00 (92.30–110.00)	< 0.001
BMI, Median (IQR)	30.10 (25.88–35.46)	27.80 (24.20–32.24)	< 0.001
WAIST, Median (IQR)	107.40 (94.93–119.50)	97.40 (87.50–108.20)	< 0.001
VAI, Median (IQR)	163.10 (91.54–267.16)	125.62 (76.96–208.98)	< 0.001
TyG, Median (IQR)	8.79 (8.37–9.28)	8.57 (8.17–9.00)	< 0.001
TG/HDL-C, Median (IQR)	94.81 (57.77–156.31)	75.86 (46.38–124.59)	< 0.001
METS-IR, Median (IQR)	1.82 (1.70–1.93)	1.71 (1.61–1.81)	< 0.001
HOMA-IR, Median (IQR)	3.11 (1.70–6.97)	2.37 (1.42–4.15)	< 0.001
*Gender, n (%)*			< 0.001
Male	278 (56)	7,603 (48)	
Female	222 (44)	8,202 (52)	
*Race, n (%)*			< 0.001
Mexican American	42 (8.4)	2,555 (16)	
Other Hispanic	36 (7.2)	1,370 (8.7)	
Non-Hispanic White	288 (58)	7,069 (45)	
Non-Hispanic Black	108 (22)	3,145 (20)	
Other Race—Including Multi-Racial	26 (4.4)	1,666 (11)	

^1^Median (IQR) or Frequency (%)

^2^Wilcoxon rank sum test; Pearson's Chi-squared test

### Evaluating the effects of metabolic indicators and Scores on AP, CHD, HF, MI using 100 machine learning algorithms

**Fig. 5:**
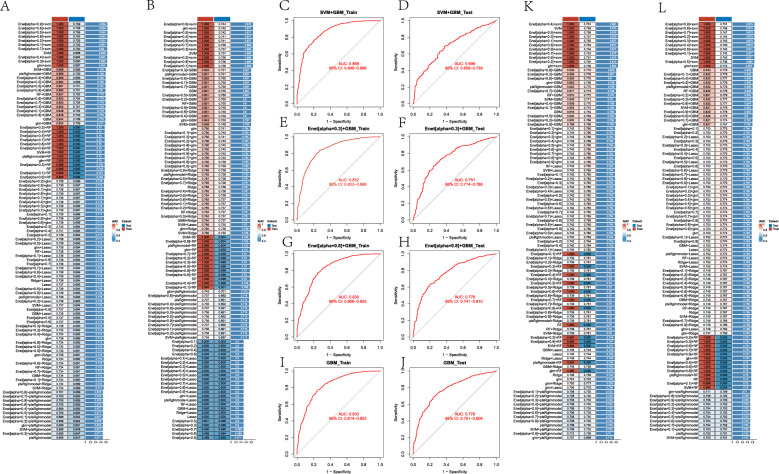
Evaluation of AUC values for AP, CHD, HF, MI prediction using 100 machine learning algorithms. **A** presents the AUC values for AP prediction using 100 machine learning algorithms. **B** shows the AUC values for CHD prediction using 100 machine learning algorithms. **C** displays the AUC value of SVM + GBM algorithm on the training set. **D** illustrates the AUC value of SVM + GBM algorithm on the testing set. **E** demonstrates the AUC value of Enet[alpha = 0.3] + GBM algorithm on the training set. **F** depicts the AUC value of Enet[alpha = 0.3] + GBM algorithm on the testing set. **G** exhibits the AUC value of Enet[alpha = 0.8] + GBM algorithm on the training set. **H** showcases the AUC value of Enet[alpha = 0.8] + GBM algorithm on the testing set. **I** shows the AUC value of GBM algorithm on the training set. **J** reveals the AUC value of GBM algorithm on the testing set. **K** represents the AUC value for HF prediction using 100 machine learning algorithms. **L** indicates the AUC value for MI prediction using 100 machine learning algorithms

To further explore the predictive effectiveness of the metabolic indicators and scores we established for AP, CHD, HF, MI, we utilized 100 machine learning algorithms to calculate the AUC values of the metabolic indicators and scores in predicting AP, CHD, HF, MI. The results showed that among the 100 machine learning algorithms, the algorithm with the highest combined AUC values for training and testing sets in predicting AP, after excluding overfitting, was SVM + GBM (Fig. 5A), with a training set AUC value of 0.868 (Fig. 5C) and a testing set AUC value of 0.699 (Fig. 5D). The algorithm with the highest combined AUC values for training and testing sets in predicting CHD was Enet[alpha = 0.3] + GBM (Fig. 5B), with a training set AUC value of 0.852 (Fig. 5E) and a testing set AUC value of 0.751 (Fig. 5F). The algorithm with the highest combined AUC values for training and testing sets in predicting HF was Enet[alpha = 0.8] + GBM (Fig. 5K), with a training set AUC value of 0.830 (Fig. 5G) and a testing set AUC value of 0.776 (Fig. 5H). Finally, the algorithm with the highest combined AUC values for training and testing sets in predicting MI was GBM (Fig. 5L), with a training set AUC value of 0.833 (Fig. 5I) and a testing set AUC value of 0.778 (Fig. 5J). To identify the metabolic scores that have the greatest impact on AP, CHD, HF, and MI, we also performed feature selection using the algorithm with the highest combined AUC values for training and testing sets in predicting AP, CHD, HF, and MI. The results revealed that METS-IR had the highest contribution among the metabolic scores for AP, CHD, HF, and MI.

## Discussion

The results of this study showed that among the six machine learning algorithms used to evaluate the effects of metabolic indicators and scores on angina pectoris, coronary disease, myocardial infarction, and heart failure, the XGBoost algorithm had the highest scores in all four conditions and is the most suitable machine learning algorithm for assessing adverse cardiovascular events. We then established the shap model of the XGBoost algorithm to explain its performance. The results showed that waist circumference was the most important metabolic indicator in angina pectoris, myocardial infarction, and heart failure, while BMI was the most crucial metabolic indicator in coronary disease. In adverse cardiovascular events such as angina pectoris, coronary disease, myocardial infarction, and heart failure, METS-IR played the most critical role in the XGBoost algorithm, with its contribution and importance surpassing the four metabolic scores of TyG, HOMA-IR, TG/HDL-C, and VAI.Furthermore, we utilized 100 machine learning algorithms to calculate the AUC values of the metabolic indicators and scores in predicting AP, CHD, HF, and MI. Subsequently, SVM + GBM, Enet[alpha = 0.3] + GBM, Enet[alpha = 0.8] + GBM, and GBM were used to perform feature selection on the metabolic indicators and scores we established. The results demonstrated that among AP, CHD, HF, and MI, METS-IR was the metabolic score with the highest contribution.

Research has shown that increased BMI significantly increases the risk of coronary disease, and triglycerides play an important mediating role in the effect of BMI on coronary disease [25]. Waist circumference, on the other hand, is an important indicator of obesity and is significantly associated with the risk of angina pectoris or myocardial infarction [26]. In the population of heart failure patients, abdominal obesity determined by waist circumference can increase cardiac compliance and inflammatory response, leading to fluid retention and exacerbating the development of heart failure [27].

Similarly, the relationship between METS-IR and heart failure has been widely studied in the XGBoost algorithm. Studies have shown a significant correlation between long-term elevated METS-IR and the high risk of coronary disease in rural populations [28], and the level of METS-IR in patients with myocardial ischemia (including angina pectoris and myocardial infarction) is significantly higher than that in non-myocardial infarction patients [29].METS-IR as an index to assess the degree of insulin resistance. In endothelial cells, insulin resistance can reduce the tyrosine phosphorylation of IRS-1 and IRS-2, decrease the activity of the PI3 K-Akt signaling pathway, and subsequently affect the activity of nitric oxide synthase and the release of nitric oxide, ultimately leading to endothelial dysfunction, decreased vasodilation capacity, and the promotion of arterial plaque formation and atherosclerosis in coronary disease patients[30, 31]. In addition, activation of the PI3 K/Akt pathway can inhibit apoptosis of myocardial cells, but under the state of insulin resistance, the PI3 K/Akt pathway is inhibited, resulting in a decrease in anti-apoptotic ability and an increased risk of apoptosis in myocardial cells, leading to injury to the coronary disease heart cells and a reduction in myocardial cells [32, 33]; activation of the PI3 K/Akt pathway can also promote cell proliferation and migration, participating in the repair and regeneration process of myocardial tissue[34, 35]. However, under insulin resistance, the function of the PI3 K/Akt pathway is inhibited, which may reduce the proliferation and migration ability of myocardial cells and hinder the repair of necrotic myocardial tissue in coronary disease patients [36, 37]. Furthermore, when insulin signaling is blocked, it can trigger intracellular inflammatory and stress responses, leading to the degradation of IκB (inhibitor) and activation of NF-κB. Activated NF-κB enters the cell nucleus, promoting the transcription of inflammatory factors, cell adhesion molecules, and apoptotic regulatory factors. In endothelial cells, the activation of the NF-κB signaling pathway mediates an increase in the expression of leukocyte adhesion molecules, enhancing the inflammatory response. The enhanced inflammatory response further induces adhesion molecule expression in endothelial cells, leading to leukocyte adhesion and infiltration of inflammatory cells. These inflammatory responses and cell infiltration promote the development of atherosclerosis in patients with myocardial ischemia [38, 39]. Additionally, in a state of insulin resistance, the balance of cell cycle regulatory factors such as cyclin-dependent kinases (CDK) and cell cycle inhibitors (CKI) may be disturbed, leading to abnormal vascular smooth muscle cell cycle and promoting abnormal cell proliferation. At the same time, interference with insulin signaling may induce the production and release of various growth factors such as platelet-derived growth factor (PDGF) and epidermal growth factor (EGF). These cell cycle regulatory factors and growth factors can stimulate the proliferation and migration of vascular smooth muscle cells, promoting the development of atherosclerosis in patients with myocardial ischemia [40, 41].

Similarly, studies have shown a positive correlation between METS-IR and heart failure, with a saturated turning point effect at 40.966, suggesting the potential of METS-IR as a key indicator for predicting heart failure [42]. METS-IR as an index to assess the degree of insulin resistance.In patients with heart failure, insulin resistance leads to a decrease in fatty acid uptake and oxidative capacity, while the uptake capacity of TG increases, resulting in the accumulation of triglycerides (TG) and fatty acids in cardiac cells. At the same time, insulin resistance also disrupts mitochondrial function, inhibits ATP production, and generates excess reactive oxygen species (ROS), damaging mitochondrial DNA and further affecting mitochondrial function [43]. Studies have also shown that oxidative stress and reactive oxygen species (ROS) produced in heart failure exacerbate the development of insulin resistance. The activation of the renin–angiotensin–aldosterone system (RAAS) and sympathetic nervous system (SNS) increases oxidative stress and ROS production, including superoxide ions (O2^-) and hydrogen peroxide (H2O2). ROS can inhibit the function of IRS-1 (insulin receptor substrate-1), leading to the blocking of insulin signaling pathways and exacerbating insulin resistance [44]. Simultaneously, in the insulin-resistant state of heart failure patients, the production of inflammatory factors such as tumor necrosis factor-alpha (TNF-α) and interleukin-6 (IL-6) increases. These inflammatory factors can activate inflammatory signaling pathways, such as nuclear factor kappa B (NF-κB) and mitogen-activated protein kinases (MAPKs) [45]. The activity of the NF-κB pathway increases during insulin resistance, leading to increased expression of inflammatory factors, activation of apoptotic pathways, increased apoptosis of myocardial cells, and damage to myocardial function. Insulin resistance also leads to enhanced activity of the MAPK pathway and its branches ERK, JNK, and p38, triggering an inflammatory response, increasing cardiac fibrosis, causing changes in myocardial structure, and reducing myocardial contractile function, exacerbating the development of heart failure [46].

METS-IR, as an index for assessing the degree of insulin resistance, plays a role in heart failure (HF) through complex molecular-level regulation mechanisms. Insulin resistance may influence cardiac cell metabolism and growth by modulating signaling pathways such as AMPK (AMP-activated protein kinase) and mTOR (mammalian target of rapamycin), thereby affecting the contraction and relaxation functions of the heart during the heart failure process [47]. In a state of insulin resistance, the AMPK signaling pathway may be overactivated or inhibited, leading to energy metabolism disorders, which can result in abnormal nucleic acid synthesis and metabolism [48]. Simultaneously, mTOR is activated through two distinct signaling pathways: one is the insulin signaling pathway (PI3 K/Akt/mTOR), and the other is the growth factor signaling pathway (Ras/Raf/MEK/ERK). Activated mTORC1 (mTOR complex 1) promotes protein synthesis, cell cycle progression, and cell proliferation. In a state of insulin resistance, the mTOR signaling pathway may be overregulated or inhibited, leading to abnormal protein synthesis and restricted cell proliferation, ultimately contributing to the development of heart failure [49].Insulin resistance affects myocardial hypertrophy and fibrosis in heart failure patients through the regulation of multiple cellular pathways. In a state of insulin resistance, the activity of the adenosine monophosphate-activated protein kinase (AMPK) signaling pathway decreases, leading to a reduction in the oxidation of fatty acids in myocardial cells and enhancing dependence on glucose uptake, ultimately causing myocardial hypertrophy. In addition, insulin resistance can promote myocardial cell growth by enhancing the activity of extracellular signal-regulated kinases (ERK) family members, exacerbating myocardial hypertrophy in heart failure patients [50, 51].

When analyzing the relationship between METS-IR and coronary heart disease and heart failure, it is necessary to consider possible metabolic abnormalities overlap and comorbidity characteristics. For example, other metabolic diseases such as obesity and diabetes may interact with insulin resistance and cardiovascular diseases, affecting the interpretation and inference of research results. In addition, the lifestyle and genetic factors of individual patients may also have an impact on the results of the study, which needs further in-depth discussion.

In this study, the biomarker model performed excellently, especially in assessing cardiovascular events such as angina, coronary heart disease, myocardial infarction, and heart failure. However, we still need to explore whether this biomarker model can serve as a universal model for the highly diverse metabolic conditions in the general population, including aspects such as stability and adaptability of the model, clinical application and feasibility, as well as potential biases and limitations.

The stability and accuracy of the biomarker model are prerequisites for it to become a universal model. We need to consider the adaptability of this model to different populations and metabolic states. For example, metabolic markers such as BMI and waist circumference may exhibit different correlations in different ethnicities, age groups, and genders. Therefore, the model's ability to personalize adjustments for different metabolic types will directly impact its effectiveness in the general population. In clinical practice, the implementation of the biomarker model needs to consider its practicality and acceptability. Whether medical teams can effectively utilize this model for patient risk assessment and intervention decisions will largely depend on the training level of clinical personnel and the cooperation level of patients. Additionally, the implementation of the model may need to be combined with existing treatment guidelines and healthcare policies to enhance its utility. Despite the outstanding performance of this biomarker model in the current research, we must also be cautious of potential biases and limitations. For example, the model may be influenced by environmental factors, lifestyle, coexisting diseases, etc., which could lead to reduced predictive ability in specific populations. Therefore, in future research, it is crucial to systematically validate the applicability of the model in different backgrounds and clinical conditions to enhance its scientific rigor and clinical significance.

## Conclusion

We conducted an in-depth analysis utilizing six machine learning algorithms, with the XGBoost algorithm emerging as the optimal binary classification machine learning algorithm in our study. This algorithm was specifically employed to evaluate the impact of metabolic indicators and scores on adverse cardiovascular events, including angina pectoris, coronary disease, myocardial infarction, and heart failure.

Through the SHAP model explanation of the XGBoost algorithm, we were able to identify waist circumference as the most crucial metabolic indicator influencing angina pectoris, myocardial infarction, and heart failure, while BMI played a pivotal role in coronary disease. Furthermore, in our evaluation of adverse cardiovascular events, including angina pectoris, coronary disease, myocardial infarction, and heart failure, METS-IR emerged as the most critical metabolic score. Remarkably, the contribution and significance of METS-IR surpassed that of other important metabolic scores such as TyG, HOMA-IR, TG/HDL-C, and VAI.

In an effort to comprehensively assess the predictive performance of the metabolic indicators and scores, we employed 100 machine learning algorithms to calculate the AUC values for predicting angina pectoris, coronary heart disease, heart failure, and myocardial infarction. Subsequently, we employed SVM + GBM, Enet[alpha = 0.3] + GBM, Enet[alpha = 0.8] + GBM, and GBM for feature selection analysis on the established metabolic indicators and scores. The results of this analysis unequivocally identified METS-IR as the metabolic score with the highest contribution among angina pectoris, coronary heart disease, heart failure, and myocardial infarction.

This comprehensive evaluation underscores the pivotal role of METS-IR as a key metabolic score in predicting adverse cardiovascular events, further highlighting its importance in the context of cardiovascular risk assessment. These findings provide valuable insights for understanding the intricate relationship between metabolic indicators and cardiovascular outcomes, contributing to the advancement of personalized medicine in cardiovascular care.

## Limitation

In this study, we have made some important findings, but there are still some limitations and future prospects that need further exploration.

First, sample selection may affect the reliability of the results. Although this study used data from the National Health and Nutrition Examination Survey (NHANES), the samples were based solely on the American population and may not fully represent patients from other countries or regions. Therefore, future research should consider validation in different populations to enhance the generalizability of the results.

Second, although we employed multiple machine learning algorithms and validated the model performance through cross-validation, the complexity of the model may lead to overfitting issues. Future research should focus on model validation to improve the effectiveness for practical clinical application.

Additionally, our study mainly focused on the association between metabolic indicators and cardiovascular events without fully considering other possible influencing factors such as lifestyle, genetic factors, and environmental factors. These factors may play a crucial role in the development of cardiovascular diseases, so future studies should incorporate more variables in the study design to comprehensively assess their impact on cardiovascular events.

Lastly, while this study emphasizes the importance of metabolic scores like METS-IR in predicting adverse cardiovascular events, future efforts should promote the use of these scores in clinical practice to enable early identification of high-risk patients and develop targeted preventive measures.

## Supplementary Information

Additional file 1.

## Data Availability

Data is provided within the manuscript.
