# Supplementary material for: Evaluating the impact of metabolic indicators and scores on cardiovascular events using machine learning
Source: Diabetol Metab Syndr. 2025 May 30;17:180. doi: 10.1186/s13098-025-01753-1 (PMC12123715; doi:10.1186/s13098-025-01753-1)
Supplement: Supplementary file 1 — Additional file 1. [file 13098_2025_1753_MOESM1_ESM.pdf]

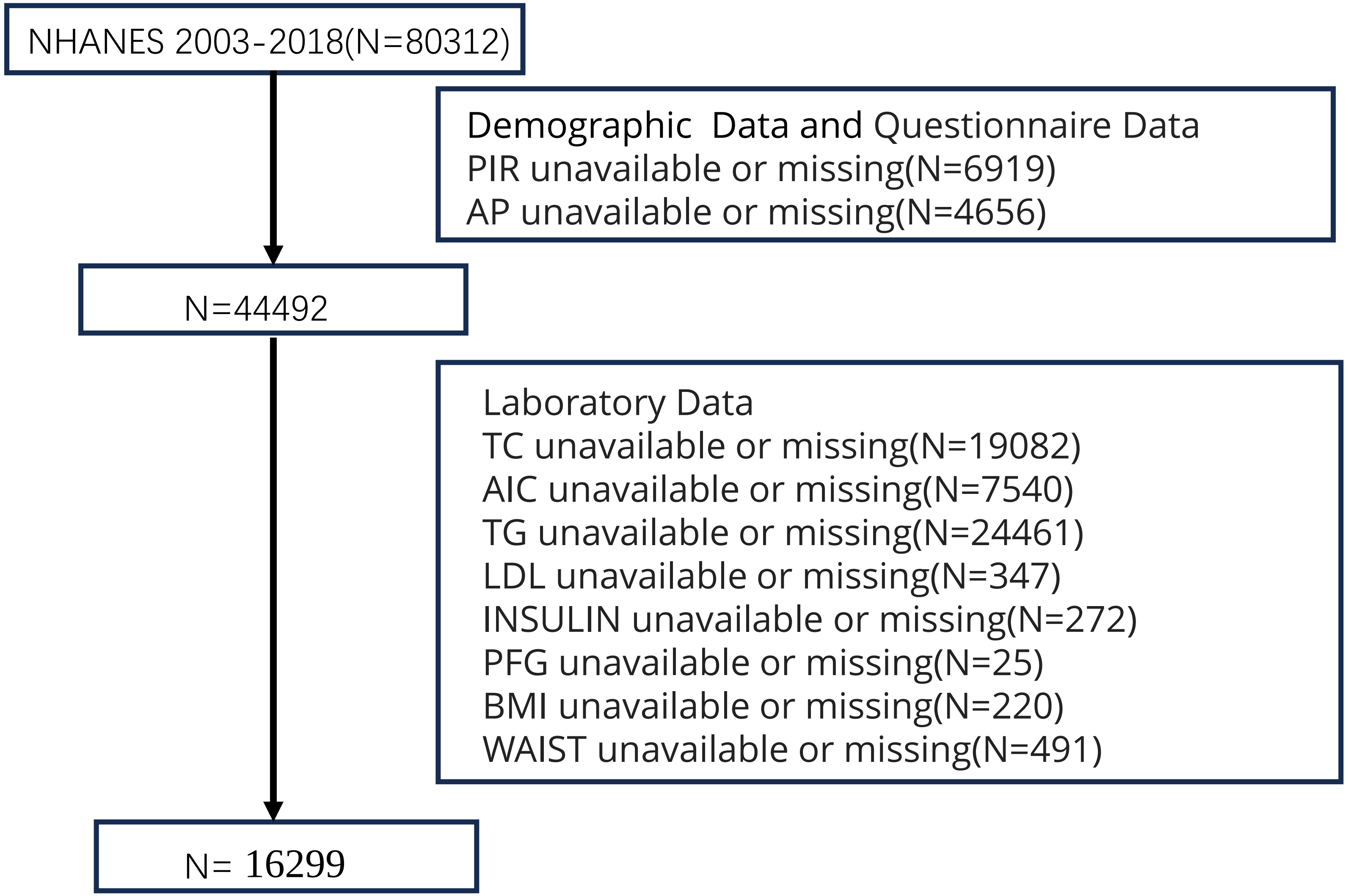
NHANES 2003-2018(N=80312)
Demographic Data and Questionnaire Data
PIR unavailable or missing(N=6919)
AP unavailable or missing(N=4656)
N=44492
Laboratory Data
TC unavailable or missing(N=19082)
AIC unavailable or missing(N=7540)
TG unavailable or missing(N=24461)
LDL unavailable or missing(N=347)
INSULIN unavailable or missing(N=272)
PFG unavailable or missing(N=25)
BMI unavailable or missing(N=220)
WAIST unavailable or missing(N=491)
N= 16299

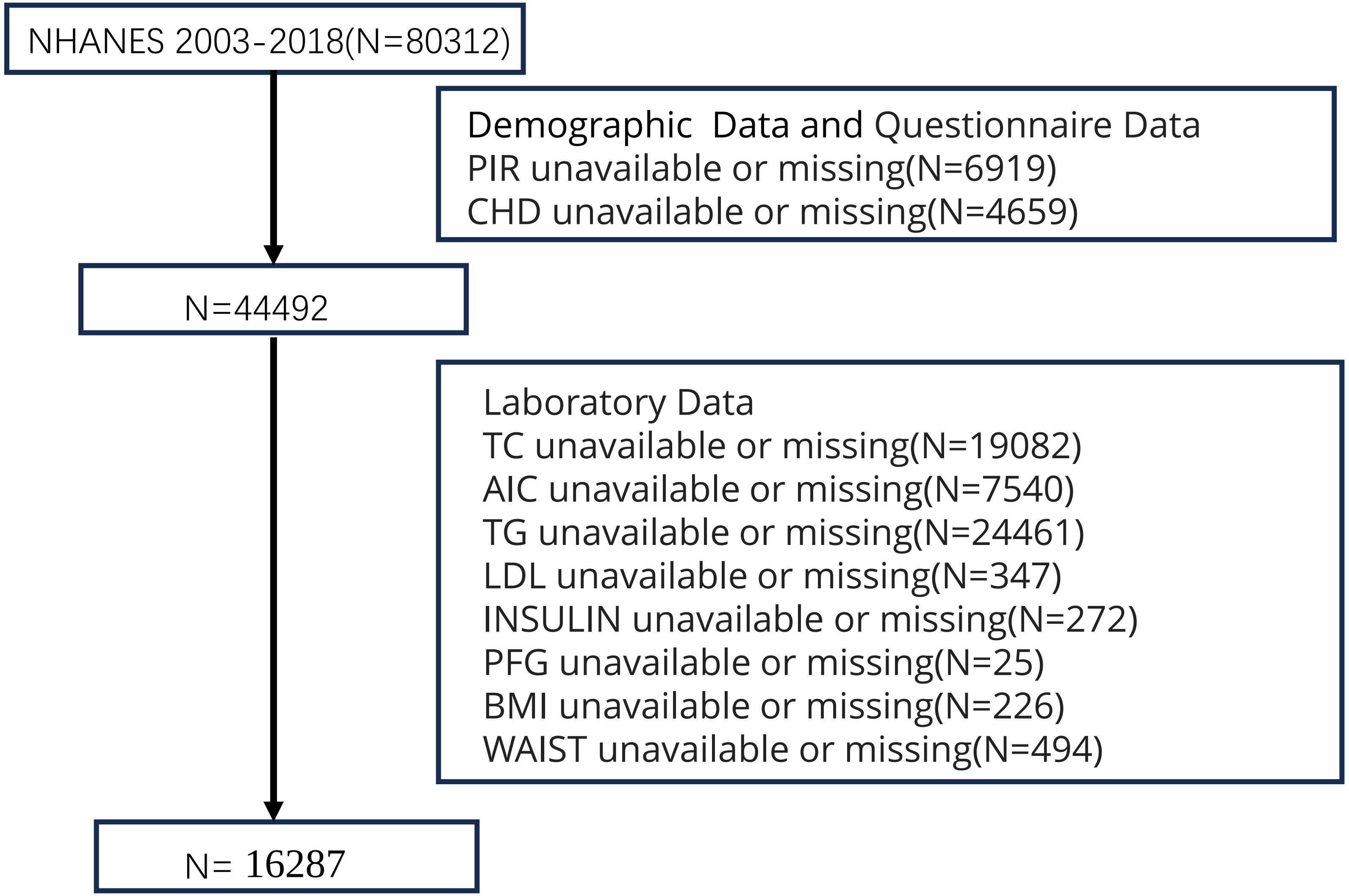
NHANES 2003-2018(N=80312)
Demographic Data and Questionnaire Data
PIR unavailable or missing(N=6919)
CHD unavailable or missing(N=4659)
N=44492
Laboratory Data
TC unavailable or missing(N=19082)
AIC unavailable or missing(N=7540)
TG unavailable or missing(N=24461)
LDL unavailable or missing(N=347)
INSULIN unavailable or missing(N=272)
PFG unavailable or missing(N=25)
BMI unavailable or missing(N=226)
WAIST unavailable or missing(N=494)
N= 16287

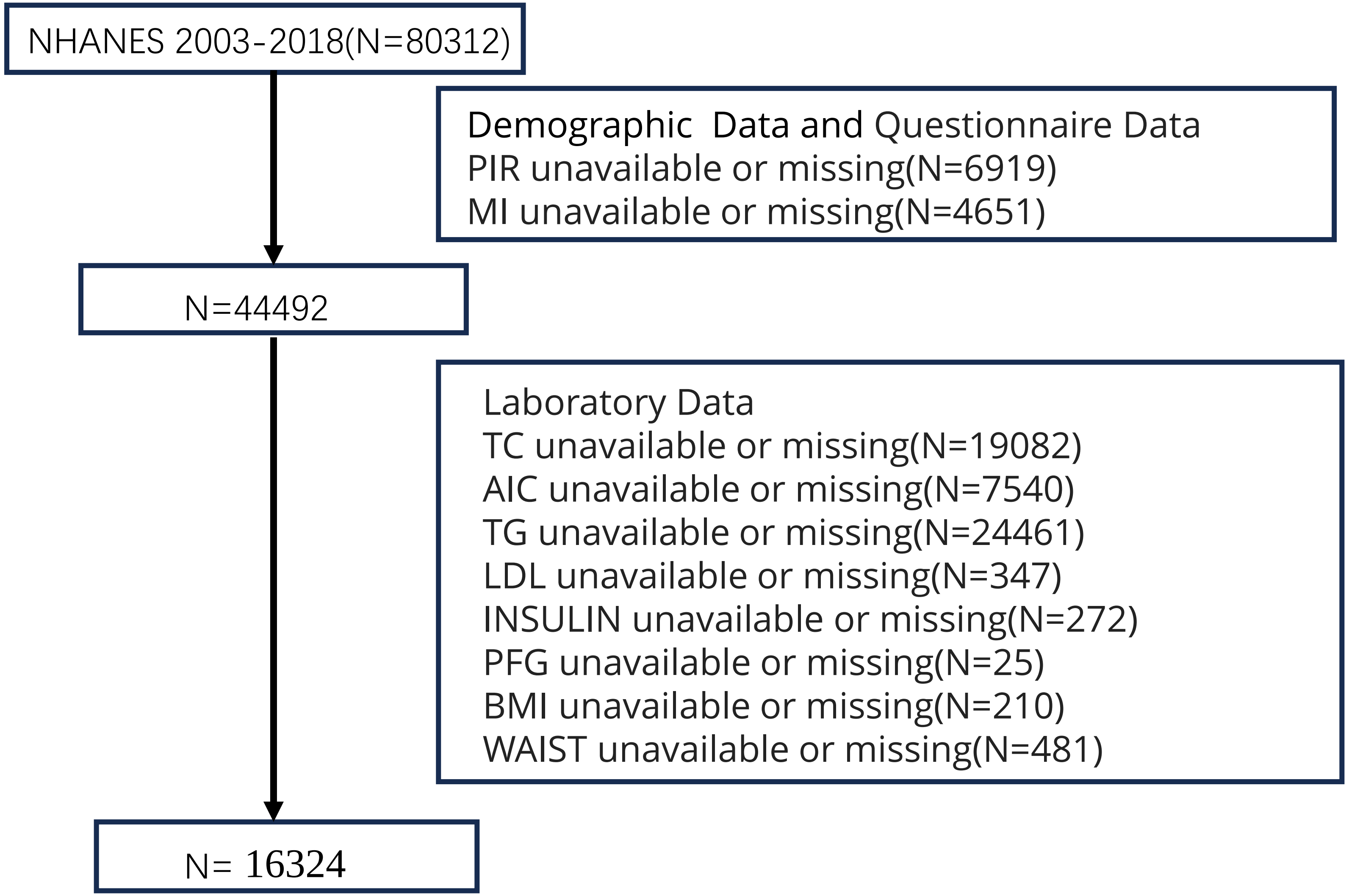
NHANES 2003-2018(N=80312)
Demographic Data and Questionnaire Data
PIR unavailable or missing(N=6919)
MI unavailable or missing(N=4651)
N=44492
Laboratory Data
TC unavailable or missing(N=19082)
AIC unavailable or missing(N=7540)
TG unavailable or missing(N=24461)
LDL unavailable or missing(N=347)
INSULIN unavailable or missing(N=272)
PFG unavailable or missing(N=25)
BMI unavailable or missing(N=210)
WAIST unavailable or missing(N=481)
N= 16324

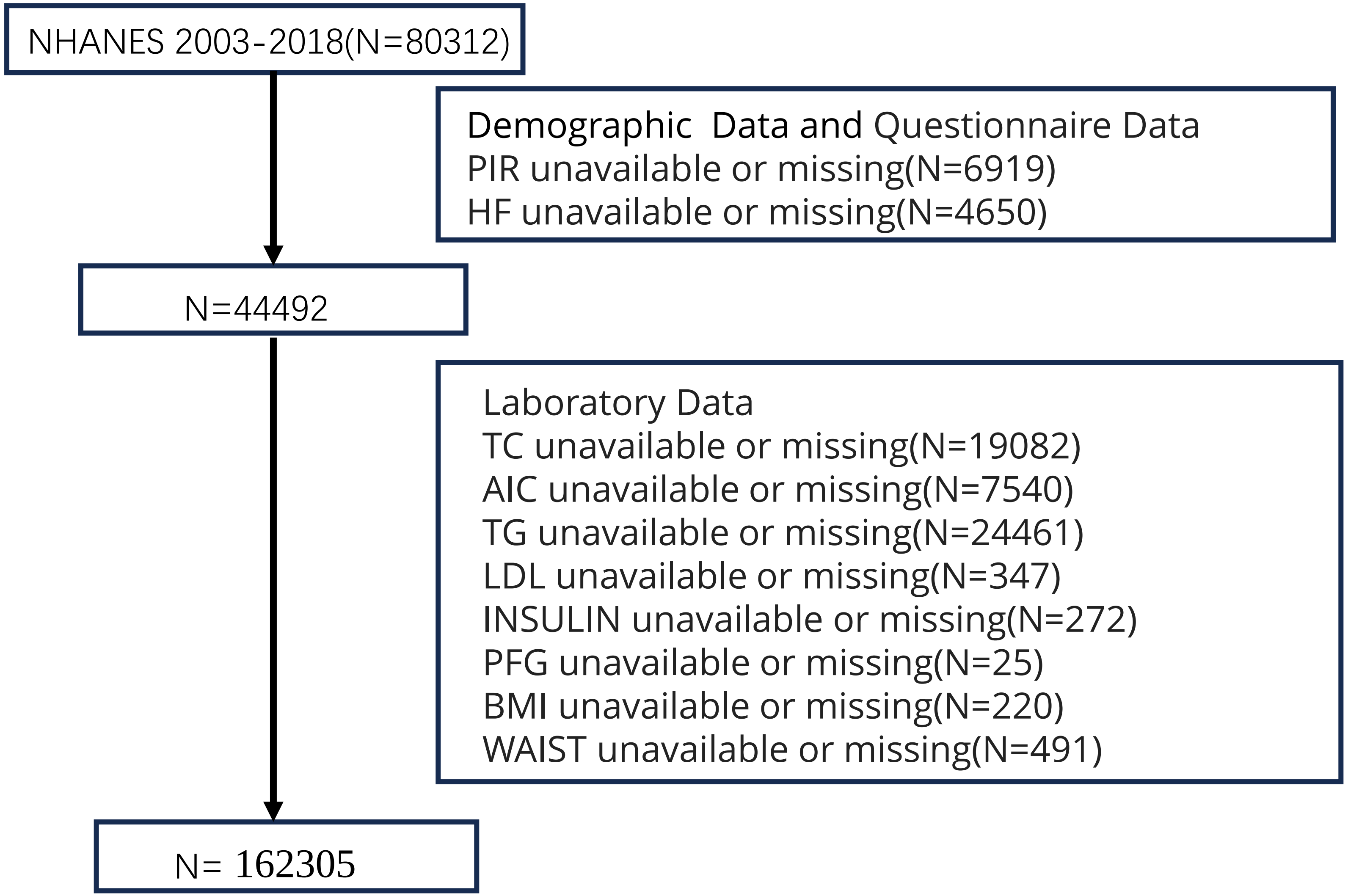
NHANES 2003-2018(N=80312)
Demographic Data and Questionnaire Data
PIR unavailable or missing(N=6919)
HF unavailable or missing(N=4650)
N=44492
Laboratory Data
TC unavailable or missing(N=19082)
AIC unavailable or missing(N=7540)
TG unavailable or missing(N=24461)
LDL unavailable or missing(N=347)
INSULIN unavailable or missing(N=272)
PFG unavailable or missing(N=25)
BMI unavailable or missing(N=220)
WAIST unavailable or missing(N=491)
N= 162305
